# Excited-State Dynamics of Room-Temperature Phosphorescent Organic Materials Based on Monobenzil and Bisbenzil Frameworks

**DOI:** 10.3390/ma13173904

**Published:** 2020-09-03

**Authors:** Kaveendra Maduwantha, Shigeyuki Yamada, Kaveenga Rasika Koswattage, Tsutomu Konno, Takuya Hosokai

**Affiliations:** 1 Faculty of Graduate Studies, Sabaragamuwa University of Sri Lanka, P.O. Box 02, Belihuloya 70140, Sri Lanka; kavindrarox@gmail.com; 2 National Metrology Institute of Japan, National Institute of Advanced Industrial Science and Technology, 1-1-1 Higashi, Tsukuba 305-8565, Japan; koswattagekr@appsc.sab.ac.lk; 3 Faculty of Molecular Chemistry and Engineering, Kyoto Institute of Technology, Matsugasaki, Sakyo-ku, Kyoto 606-8585, Japan; syamada@kit.ac.jp (S.Y.); konno@kit.ac.jp (T.K.); 4 Department of Engineering Technology, Faculty of Technology, Sabaragamuwa University of Sri Lanka, P.O. Box 02, Belihuloya 70140, Sri Lanka

**Keywords:** room temperature phosphorescence, organic molecule, excited state dynamics, time-resolved photoluminescence spectroscopy, photostability

## Abstract

Room-temperature phosphorescent (RTP) materials have been attracting tremendous interest, owing to their unique material characteristics and potential applications for state-of-the-art optoelectronic devices. Recently, we reported the synthesis and fundamental photophysical properties of new RTP materials based on benzil, i.e., fluorinated monobenzil derivative and fluorinated and non-fluorinated bisbenzil derivative analogues [Yamada, S. et al., Beilstein J. Org. Chem. 2020, 16, 1154–1162.]. To deeply understand their RTP properties, we investigated the excited-state dynamics and photostability of the derivatives by means of time-resolved and steady-state photoluminescence spectroscopies. For these derivatives, clear RTP emissions with lifetimes on the microsecond timescale were identified. Among them, the monobenzil derivative was found to be the most efficient RTP material, showing both the longest lifetime and highest amplitude RTP emission. Time-resolved photoluminescence spectra, measured at 77 K, and density functional theory calculations revealed the existence of a second excited triplet state in the vicinity of the first excited singlet state for the monobenzil derivative, indicative of the presence of a fast intersystem crossing pathway. The correlation between the excited state dynamics, emission properties, and conformational flexibility of the three derivatives is discussed.

## 1. Introduction

In the last decade, room-temperature phosphorescent (RTP) materials have drawn intense interest, owing to their potential uses in medical applications, such as bio-imaging, security applications, including security inks for information encryption and anticounterfeiting measures, as well as photosensitizers, in emergency signage, in geochemical dating, in advanced turbo-machinery, for oxygen measurements inside and outside living organisms, and for electronic applications, such as data storage, logic gates and organic light-emitting diodes (OLEDs) [1,2,3,4,5,6,7]. Thus far, heavy-metal-based inorganic compounds and organometallic materials have proven to be the most efficient RTP material. For instance, tris [2-phenylpyridinato-*C*^2^,*N*]iridium(III) has been reported to have a high RTP quantum yield and was investigated for its use as a highly efficient green light emitter in OLEDs [1]. Due to the presence of the Ir atom, the heavy metal effect leads to the large spin–orbit coupling (SOC) for the Ir compound, which results in the fast rate of the spin-flip processes from the first excited singlet state (S_1_) to the first excited triplet state (T_1_) (i.e., the intersystem crossing (ISC)) and from T_1_ to the ground state (S_0_) with radiative emissions (i.e., phosphorescence). However, there are major drawbacks in RTP materials containing heavy metals, including high costs, limited resources, and high toxicity. Hence, metal-free organic materials that can replace RTP materials containing heavy metals are in demand.

**Figure 1:**
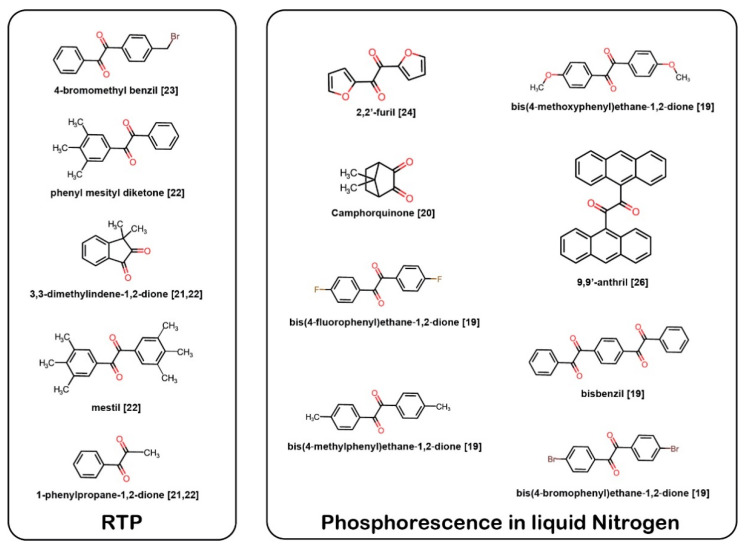
α-dicarbonyl molecules such as benzil and benzil derivatives reported to emit room-temperature phosphorescence (RTP) or phosphorescence at 77 K.

Since the first observation of the room-temperature afterglow of the metal-free organic luminophore carbazole in crystalline form in 1978 by Bilen et al., the search for efficient metal-free RTP materials and their applications has drawn significant interest [8,9,10,11,12,13,14,15,16,17]. An important RTP molecule is benzil, α-dicarbonyl (a molecule with two carbonyl (C=O) groups on neighbouring carbon atoms). Similar to the heavy-metal effect, the incorporation of carbonyl groups in organic molecules tends to induce greater SOC by lowering the S_1_-T_1_ energy gap according to the orbital selection rule between the initial and final state upon photoexcitation—that is, the El-Sayed rules [18,19]. Benzil, benzil derivatives, and dicarbonyl compounds (Figure 1) have been reported to emit either RTP or phosphorescence at liquid nitrogen temperatures [19,20,21,22,23,24,25,26]. Among these compounds, benzil has been the subject of extensive investigations in the field of photochemistry because conformational changes upon photoexcitation have been reported [18,24,25,27,28,29]. Benzil in S_0_ has a twisted geometry, and upon photoexcitation, the geometry reorients into a trans-planar (TP) geometry. Benzil in the TP geometry is reported to emit light from both excited singlet and triplet states. The conformational flexibility is known to enhance the SOC by the twisting around the bond between the two carbonyl groups, which reduces the energy gap between excited singlet and triplet states [18,19]. As a result, benzil shows efficient ISC with a high quantum yield of 0.92 (in solid form) [30]. Thus, in this study, we investigated the photochemistry of novel derivatives based on the benzil structure.

**Figure 2:**
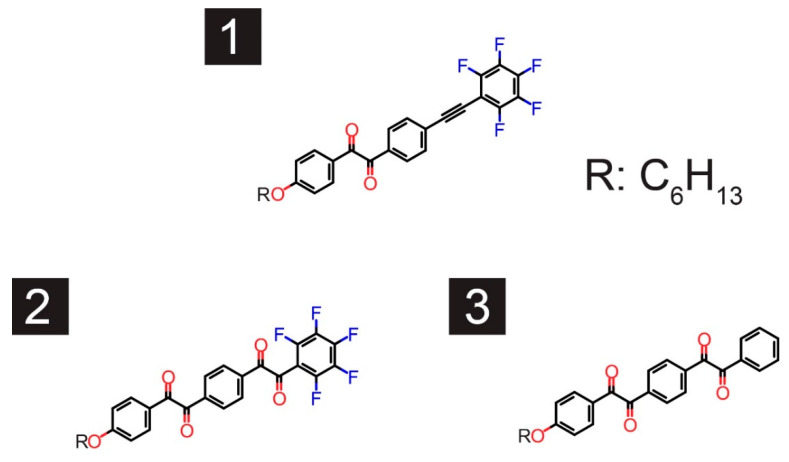
Fluorinated monobenzil (**1**), fluorinated bisbenzil (**2**) and non-substituted (**3**) bisbenzil derivatives.

Very recently, we reported the synthesis of new derivatives based on the benzil framework (Figure 2), as well as their steady-state photophysical properties [31]. Fluorinated monobenzil **1** and fluorinated (or non-substituted) bisbenzil **2** (and **3**), which are synthesized by a facile PdCl_2_/dimethyl sulfoxide (DMSO) oxidation protocol from common bistolane derivatives, are capable of emitting RTP. The photoluminescence quantum yields (PLQYs), recorded for the derivatives in toluene solutions, are approximately 2% for **1** and <1% for **2** and **3**. The absorption peaks at approximately 400 nm, which is the maximum absorption wavelength of the derivatives, are attributed to an n-π^*^ transition based on theoretical calculations. We suspected that the fluorination of one side of the aromatic ring of the benzil derivatives may retard the ISC, leading to weaker RTP. In this study, we studied the excited-state dynamics of benzil derivatives using time-resolved PL spectroscopy to understand their photophysical properties. In addition, we investigated the photostability of **1**–**3** using an Xe discharge lamp. The fundamental question is how do the side groups of the benzil derivatives affect the conformational flexibility and emission of RTP by comparison with benzil? In addition, we aimed to uncover the role of the monobenzil and bisbenzil structures, as well as the effect of the fluorination of the side group, on the low PLQY of these compounds.

## 2. Materials and Methods

All benzils were synthesized from the corresponding bistolane derivatives, following literature procedures [31], and were purified by column chromatography using a mixed solvent of hexane and EtOAc as an eluent, followed by recrystallization from hexane before use. Spectroscopic grade toluene (FUJIFILM Wako Pure Chemical Corporation; Osaka, Japan) was used as the solvent. Before preparing sample solutions, the water contaminating the solvents was removed using molecular sieves (Merck, 105704; New Jersey, NJ, USA). For all measurements, quartz cuvettes with 10 mm path lengths were used. To avoid the undesirable entry of ambient light, the sample cuvettes filled with the solutions were covered with aluminum foil. Steady-state ultraviolet-visible (UV-VIS) absorption spectra were obtained using a UV-3100 spectrometer (Shimadzu; Kyoto, Japan), whereas steady-state PL spectra were measured using a LS50B fluorescence spectrometer (Perkin Elmer; Waltham, MA, USA) at an excitation wavelength of 290 nm via an Xe discharge lamp (20 kW for 8 µs duration, and pulse width at half height <10 µs) with a long-path 320 nm filter set at the detection side. For time-resolved PL measurements using single photon counter, we used a FluoroCube (Horiba; Kyoto, Japan). For the time-correlated single photon countering (TCSPC) method, a 342 nm pulsed LED with a pulse width: 1 ns (Horiba, NanoLED™; Kyoto, Japan) was used, and for the multichannel scaler (MCS) method, a 355 nm pulsed LED (Horiba, SpectraLED™; Kyoto, Japan) was used. In addition, the third-harmonic wavelength at 355 nm of a EKSPLA PL 2210 series diode-pumped picosecond kilohertz-pulsed Nd:YAG laser (pulse width 28 ps ± 10%), together with a 12-bit oscilloscope (Teledyne Lecroy, Wavepro404HD; Chestnut Ridge, NY, USA), was used to measure the RTP lifetime. To measure the temperature-dependent PL spectra, sample solution cells were immersed directly in liquid N_2_ or set in a liquid N_2_ cryostat (Unisoku Co., Ltd., USP-203; Osaka, Japan). The solution concentration was kept the same for all the samples. For N_2_ purging of the sample solutions, pure N_2_ gas (purity: 99.99995% (6N)) was used.

Theoretical calculations based on density functional theory (DFT) and time-dependent DFT (TD-DFT) were conducted for single molecules of **1–3** using the Gaussian 16 package. The optimized geometries of **1–3** were obtained for S_0_ and S_1_. Using the S_1_ geometry, the excitation energies of both S_n_ and T_n_ levels (*n* = 1–10) were estimated. All the calculations were performed at the M06-2X/6-31g (d) level of theory.

## 3. Results and Discussion

### 3.1. Photostability

**Figure 3:**
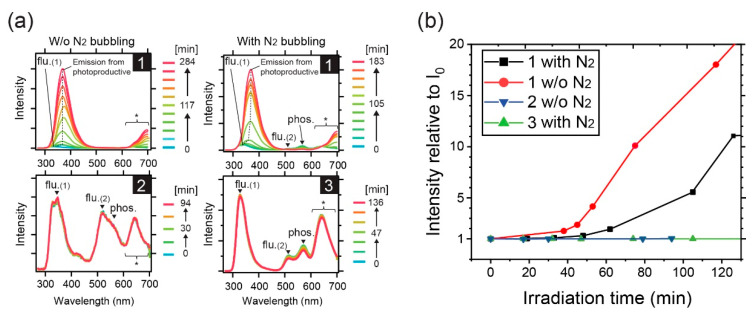
Photostability of **1−3** in toluene solutions. (**a**) Irradiation time dependence of PL spectra of **1−3** derivatives. The irradiations were conducted continuously to **1** and **2** solutions prepared w/o N_2_ bubbling, and **1** and **3** solutions with N_2_ bubbling. Asterisk (*) is the region of second order diffracted light of flu.(1) or photo-productive emission band. (**b**) Time evolution of the intensity ratio at the peak at around the flu.(1) bands (dashed line in Figure 3a) started from the initial peak intensity (*I*_0_).

First, we discuss the photostability of **1−3** in toluene based on steady-state measurements. Figure 3a shows the irradiation-time-dependent PL spectra of **1−3** in toluene obtained using a 290 nm Xe-discharge lamp. Here, **1** was measured with and without (w/o) N_2_ bubbling to investigate the photostability of both excited singlet and triplet states of the compound, respectively. For **2** and **3**, N_2_ bubbling was performed only for **3** because both **2** and **3** showed similar PL patterns despite the presence of a fluorinated phenyl ring in **2**. The initial spectra of **1** (*t* = 0 min, fresh sample) are shown in Figure S1 (see Supplemental file). The first fluorescence band (flu.(1)) peaked at 340−360 nm, the second fluorescence band (flu.(2)) peaked at approximately 520 nm, and the phosphorescence band (phos.) peaked at around 560 nm, as previously reported [31], and could be ascribed to an emission from S_2_ for flu.(1), S_1_ for flu.(2), and T_1_ for phos. bands. These bands were typical of the benzil moiety [30,32], but not tolane [33,34,35,36,37]. An emission from higher excited states implied anti-Kasha behavior, and many molecules were known to exhibit the emission [13,38,39]. It is worth noting that we confirmed that the flu.(2) band was not a thermally activated delayed fluorescence band for 2, as has been reported for benzil in imidazolium ionic liquids [29] (see Figure S2). Additionally, no excitation wavelength dependence was seen for the flu. (2) band (Figure S3). The absorption spectra of the fresh (irradiation time (*t*) = 0 min) and the final sample of **1** solution are shown in Figure S4, where the initial absorbance of the maximum absorption band at approximately 313 nm decreased by 15% after the final PL measurement. While the wavelength of the excitation light (290 nm) for the PL measurements was far from the absorption maximum wavelength at approximately 400 nm, it was chosen to excite the molecules to higher excited singlet states, enabling the detection of both flu.(1) and flu.(2) bands.

In Figure 3a, it can be seen that the irradiation of **1** by light at the excitation wavelength of 290 nm caused a continuous change in the PL intensity and peak position to a redder region, unlike **2** and **3**. For **1**, the flu.(2) and phos. bands observed after N_2_ purging decreased in intensity, whereas flu.(1) gradually increased. The spectral change was also observed irrespective of the N_2_ purging, although the change was much less significant with N_2_ purging. However, when the excitation wavelength was changed to 355 nm, the photo-induced increase in the intensity at around flu. (1) band of **1** was strongly suppressed (Figure S5). Clearly, **2** and **3** possessed much higher photostability than **1**, and the spectral change of **1** was most likely caused by a chemical reaction of the excited singlet states of **1**; otherwise, a stronger change would be expected to occur after N_2_ purging, owing to long-lived excited triplet states. For **1**, the weakening of the flu. (2) and phos. bands, which were attributed to the n-π^*^ transition of the benzil moiety, indicated chemical changes in the benzil portion. The increase in intensity at the shorter wavelength of flu. (1) band peak was attributed to an emission from photo-production. From the observation of the much higher photostability of **1** by the irradiation with 355-nm light, it was suggested that the reaction occurs through higher excited singlet states. Usually, higher excited states, such as S_2_ and T_2_, are rapidly deactivated via nonradiative routes to the corresponding lower excited states of S_1_ and T_1_ on a picosecond timescale, which is the so-called Kasha’s rule, and thus, are less reactive. However, because RTP was observed for **1−3**, the peculiar behavior in the higher excited states was understandable and will be explained later in the time-resolved PL spectra.

### 3.2. Excited-State Dynamics

**Figure 4:**
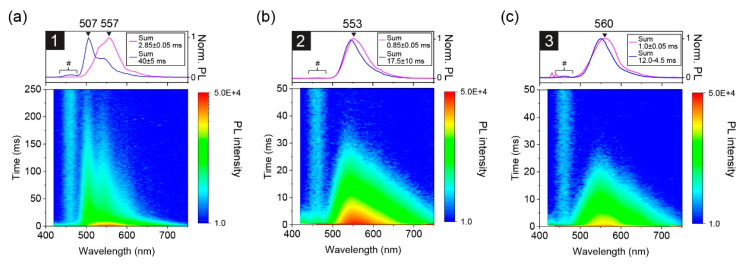
Time-resolved PL spectra of (**a**) **1**, (**b**) **2**, and (**c**) **3** at 77 K measured using the multichannel scaler (MCS) method: (top) Intensity summed PL spectra taken at time regions depicted in each graph legend, (bottom) contour map of the time-resolved results. The excitation light source was a pulsed LED at 355 nm. The region marked with a hash (#) is a background feature attributed to scattering by the sample cell containing solvent.

After investigating the photostability of **1−3** in toluene, time-resolved PL measurements were performed; there was a negligible change in the absorption spectra after the measurements was confirmed. Figure 4a–c show time-resolved millisecond PL spectra of **1−3** in toluene measured at 77 K. Theoretical curve fitting was performed to estimate the time constant (*τ*) of the observed bands (Figure S6), and the results are summarized in Table S1. Initially, an intense band was observed at around 555 nm for all the samples. The peak wavelength of the band is matched to that of their RTP band, indicating the emission from T_1_. Subsequently, their PL spectra changed gradually. For **1**, an additional band appeared at a shorter wavelength of 506 nm with vibrionic progression. Similar behavior was reported for benzil in ethanol at 77 K [32]. The high-energy phosphorescence band was not observed for **2** and **3**, and it survived much longer than the T_1_ band that was observed for all the samples. These results indicate the existence of a higher excited triplet state for **1**, that is, T_2_. For **2** and **3**, only a slight narrowing of the initially observed T_1_ emission band was identified. This can be explained by a uniform change in the molecular conformation distribution because the benzil moiety can twist during the relaxation process [18]. Importantly, the lifetime of T_2_ was longer than that of T_1_, meaning that the internal conversion (IC) from T_2_ to T_1_ was strongly suppressed at 77 K. Another point is that the peak position of the T_2_ band was almost consistent with the S_1_ band, that is, flu. (2) at room temperature, indicating a negligible energy gap (<0.01 eV) between S_1_ and T_2_. This was supported by theoretical calculations, vide infra. The very small, almost negligible, gap led us to expect a fast ISC for **1** between S_1_ and T_2_, according to the energy gap law.

**Figure 5:**
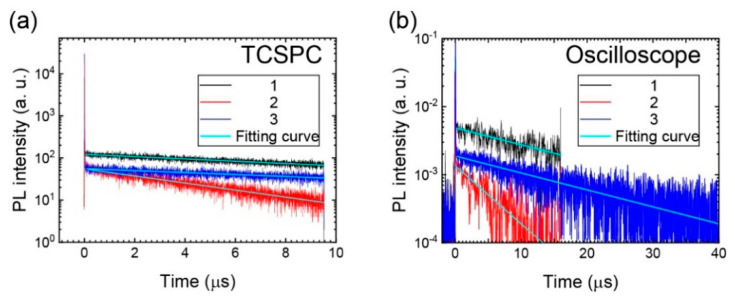
RTP lifetime measurements of **1−3** in solutions after N_2_ purging using (**a**) time-correlated single photon countering (TCSPC) (excitation wavelength: 342 nm) and (**b**) 12-bit oscilloscope (excitation wavelength: 355 nm).

The observation of a longer T_2_ lifetime compared to that of T_1_ at 77 K (Figure 4a) implies anti-Kasha behavior in **1** [38,39,40]. This suggests that **1** shows different RTP behavior to **2** and **3**. However, as we characterized the RTP time profiles under nearly identical absorbance conditions for the excitation light (see below), the existence of T_2_ in **1** was revealed to affect the ISC efficiency, but not the RTP lifetime. Figure 5a,b show the transient PL results of the RTP band measured by TCSPC and a 12-bit oscilloscope, respectively. The former was conducted first and the latter was performed to confirm the TCSPC data because our TCSPC systems had difficulty measuring the measurement time region with sufficient time resolution for **1** and **3**. A single decay exponential function was successfully used for curve fitting of the time profiles of RTP of both the measurement systems; *τ* of RTP (*τ*_RTP_) was estimated to be 16.9 µs (by TCSPC)/17.5 µs (by oscilloscope) for **1**, 5.2 µs/5.2 µs for **2**, and 18.8 µs/17.5 µs for **3.** Compounds **1** and **3** exhibited relatively large and very similar *τ*_RTP_ values compared to **2**. As **2** and **3** only differed by the substituted F atoms on the phenyl side group, we inferred that fluorination might decrease the RTP lifetime. The amplitude of **1** was stronger than **3** by a factor of 3.6, but the lifetimes were very similar. This implies a faster ISC in **1** than **3**, plausibly because T_2_ is very close to S_1_. The small difference in the amplitude between **2** and **3**, which differ by a factor of 1.4, suggests a similar ISC process for the two derivatives, but the T_1_ lifetime was longer for **3** than **2**, which results in a more intense RTP emission in non-fluorinated **3**.

**Figure 6:**
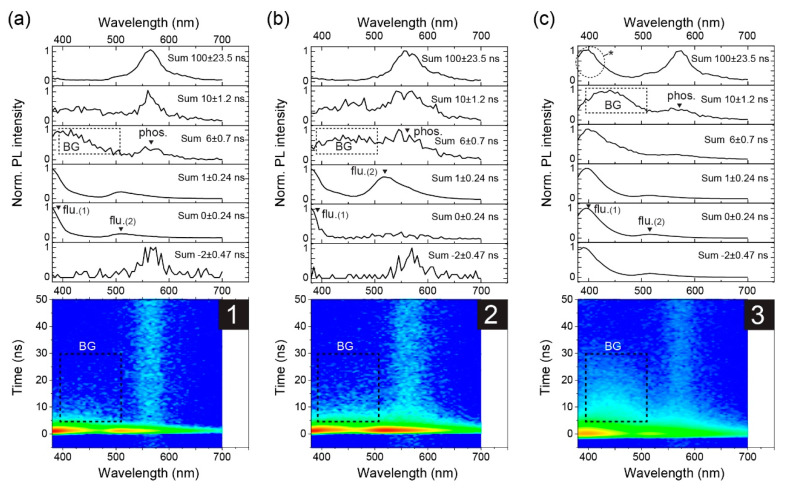
Time-dependent PL spectra of (**a**) **1**, (**b**) **2**, and (**c**) **3** in toluene solutions with N_2_ bubbling measured by TCSPC: (top) Normalized PL spectra selected at each time region, (bottom) contour map of the time-resolved results. The square region with the broken line [denoted to BG (background)] in (**c**) is the scattered light from the sample solutions. The circle region in (**c**) noted by * is a characteristic of our TCSPS setup (see Figure S7). The repetition rate of the excitation laser is 1 MHz. The RTP bands seen at -2 ns are caused by the high repetition rate close to the microsecond lifetime of RTP.

Finally, we present the room-temperature time-resolved nanosecond PL spectra of **1−3** in toluene solution. The TCSPC spectral data in Figure 6a–c show the simultaneous decay of flu.(1) and flu.(2) bands for **1−3**. This anomalous result suggests that IC from S_2_ to S_1_ and radiative decay from S_2_ to S_0_ are competitive processes for **1−3**, unlike non-RTP compounds. Due to the limited time resolution of our apparatus, we could not distinguish the IC process from the data, that is, the decrease in S_2_ and increase in S_1_, implying that S_1_ decays very fast compared to S_2_, and the IC restricts the decay time of S_1_. The values of *τ* for the flu.(1) and flu.(2) band peaks estimated by curve fitting (see Figure S8) were similar for each compound, but there was a chemical structure dependence; *τ* was shorter in the order of **1** (0.48 ns) < **2** (0.56−0.67 ns) < **3** (0.79−0.95 ns). Note that the value of 0.48 ns was almost the same as the *τ* of scattered light of the excitation light pulse of 0.45 ns, indicating that the presence *τ* of **1** may be the upper limit. Remarkably, the *τ* of **1** was significantly shorter than that of **3**, despite their similar *τ*_RTP_ values, as discussed in the previous section. These results imply that the fluorination at the side phenyl group of the benzil framework controls the radiative decay of the singlet-excited state (but not ISC) to the excited triplet state. Again, the ISC rate of **1** was different from those of the others because of the presence of the T_2_ state.

As mentioned in the introduction, excited α-dicarbonyl derivatives have been reported to be photoisomerised from a *cis*-skewed geometry in S_0_ to a TP geometry in excited S and T states [32]. However, a recent work by Bhattacharya et al. revealed that, depending on the excitation wavelength, emissions from S_1_ and S_2_ can be attributed to the skewed form of benzil, whereas S_1_ also fluoresces from the TP conformer [30]. In the present study, the observation of both S_1_ (flu.(2)) and S_2_ (flu.(1)) emissions suggest that the geometries of **1–3** in the excited singlet state are *cis*-skewed or *cis*-skewed-like, which is not a perfect TP geometry. Further, Roy et al. studied the emissions of benzil in ethanol solution at 77 K and observed a phosphorescence peak at around 530 nm. Upon increasing the temperature (to just below the melting point), a second phosphorescence band emerged at approximately 570 nm, which was ascribed to the emission originating from a coplanar (TP) geometry of the excited triplet state that grows from the relaxation of the skew geometry in the excited triplet state. Based on this result, we could expect that the TP geometry is formed in **1**–**3** at 77 K and room temperature, but only **1** can simultaneously maintain the unrelaxed skew geometry at 77 K.

**Figure 7:**
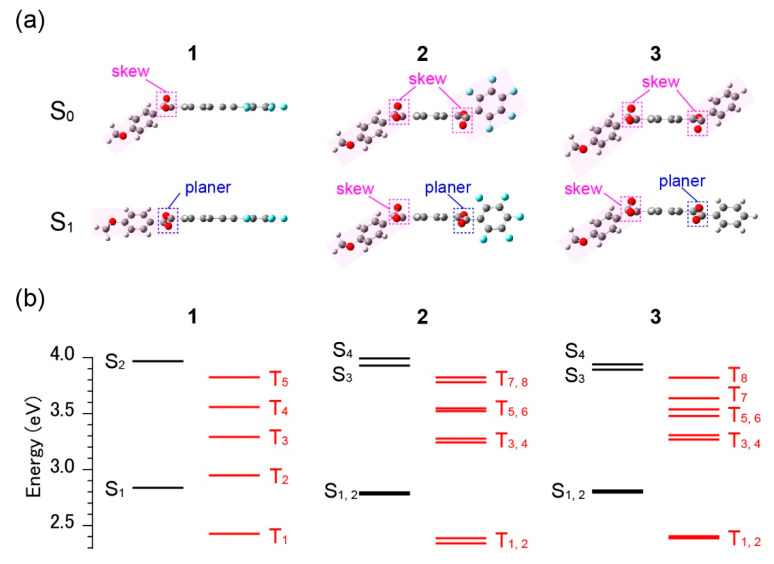
Theoretical calculations for **1–3**. (**a**) Optimised conformations at the S_0_, and S_1_ levels. (**b**) Energy level diagram of some excited S_n_ and T_n_ levels. All calculations were conducted at the M06-2X/6-31Gd level of theory. The calculated excitation energy, wavelength and oscillator strength are summarized in Table S2.

To verify the above hypotheses, we conducted theoretical calculations of **1–3** using DFT and TD-DFT. Figure 7a shows the molecular geometries of **1–3** optimised for S_0_ and S_1_ states. All the derivatives exhibit a skew geometry of the benzil moieties in S_0_. Owing to the skewed geometry, the side phenyl groups are significantly bent away from the plane of the central phenyl rings (see the pink square in Figure 7a). In the S_1_ geometry, however, the conformation of the dicarbonyl group is more planar in **1** and the side phenyl ring is close to the plane of the central phenyl ring. For **2** and **3**, the geometries of the S_1_ states are the same as for **1**, and only the dicarbonyl group connected to the other side of the phenyl with no alkoxy group becomes planar—the geometry of other dicarbonyl groups with alkoxy phenyl rings remain unchanged. A close inspection of **2** reveals that the planarity of the side perfluorinated phenyl ring relative to the central phenyl ring is lower than that of the non-substituted phenyl ring of **3**. This is an effect of the greater steric bulk of **2** compared to **3**.

In contrast, there are distinct differences in the energy level diagram in Figure 7b. In Figure 7b, only **1** possesses a T_2_ level in the vicinity of S_1_ (separation of approximately 0.1 eV). For **2** and **3**, the T_2_ levels are very close to T_1_, which is approximately 0.4 eV below S_1_. Further, the next T_3_ level is also above S_1_ by the same value. As previously mentioned, the close vicinity of T_2_ to S_1_ was expected for **1** alone, based on the time-resolved PL spectra measured at 77 K shown in Figure 4a. This supports our consideration that fast ISC occurs only for **1** according to the energy gap law because, for **2** and **3,** there is no T_n_ level close to S_1_ and the energy gap between S_1_ and T_1_ is the same (approximately 0.4 eV) for all derivatives. The latter fact, where S_1_ and T_1_ have energies within <0.05 eV for the three derivatives, is consistent with the fluorescence and phosphorescence spectra and their dynamics, which are not drastically different for **1–3**. We note that the discussion here is based on the energy gap between the corresponding excited S and T states. A complete understanding of the ISC efficiency also requires SOC constants between the states, which, in principle, is theoretically available [13].

Finally, we discuss the correlation between the dynamics and PLQY of **1–3**. Previously, we reported that the PLQY of **1** in toluene measured after N_2_ purging was 1.8%, whereas those of **2** and **3** under the same conditions were less than 1%. These results indicate that most of the excited states of **1–3** is deactivated non-radiatively to S_0_. In this sense, the high PL efficiency, particularly, the high RTP efficiency, must result from the strong suppression of the non-radiative decay process. The fact that RTP is observable because of the fast ISC (<1 ns) suggests that the excited singlet states are mostly converted to excited triplet states. This is similar to, for example, the nearly 100% triplet yield efficiency from the excited singlet state for benzophenone, whose ISC rate constant has been reported to be 9 to 16 ps^−1^ in solution [41]. Benzil has also been reported to have *τ* in the sub-picosecond to several tens-of-picoseconds regime in various solvents [25] and have a high triplet yield of 0.92 (in solid form) [30]. For **1–3**, it is likely that the decrease in conformational flexibility at the dicarbonyl group suppresses the ISC rate compared to only benzil. This is obvious for **2** and **3**, which have bulky substituents on one side of the benzil moiety that restrict the twisting motion of the other side of the benzil moiety, thus further reducing their slow ISCs compared to that of **1**. The role of fluorination of one side of the phenyl group connected to benzil is not clear at this moment. However, fluorination does not seem to help enhance the ISC or strengthen the PLQY, although the F atom is certainly heavier than the H atom. Therefore, the electronic effect of the F atom on the bisbenzil framework is not significant. In other words, the heavy metal effect does not apply to the derivatives. The conformational change in the excited triplet states should probably be considered because a clear change in conformation from the S_1_ state to the ground triplet state was only observed for **2** by theoretical calculations (Figure S9). To understand this further, the direct estimation of excited triplet states by transient absorption spectroscopy, as well as determination of the proper decay constant of excited singlet states, which was limited in the present study because of the insufficient time resolution of our apparatus, is required. Such investigations also help to draw a kinetic scheme of **1–3**. For the realization, a ratio of fluorescence and RTP ratio in PLQY, PLQY at 77 K, the exact *τ* value of flu.(1) and flu.(2) bands are also demanded.

## 4. Conclusions

We investigated the excited state dynamics and photostability of monobenzil and bisbenzil derivatives (**1–3**) in toluene. Monobenzil derivative **1** exhibited strong degradation upon irradiation with 290 nm light. Time-resolved PL studies uncovered the following findings.

1. The lifetime of RTP for the present benzil derivatives is the microsecond regime.
2. The monobenzil derivative possesses a longer lifetime and more intense RTP emission than the bisbenzil derivatives.
3. Fluorination at one side of the phenyl groups of benzil does not significantly affect the behavior of the excited triplet state but shortened the lifetime of the excited singlet state.

From the experimental and theoretical results, the distinct characteristics of the monobenzil derivative can be explained by the T_2_ state having almost the same energy as S_1_. For efficient RTP materials containing a benzil framework, the attachment of bulky substituents may be undesirable because they suppress the conformational flexibility at the dicarbonyl portion, which is key to the efficiency of the ISC of benzil derivatives.

## Supplementary Materials

The following are available online at https://www.mdpi.com/1996-1944/13/17/3904/s1, Figure S1: Steady state PL spectra of fresh **1** solutions (*t* = 0 min), taken from Figure 3a, Figure S2: Temperature dependence of PL spectra of **2** after N_2_ bubbling, Figure S3: Excitation wavelength dependence of flu.(2) band peak of **1** and **2**, Figure S4: Absorptions spectra of **1** in toluene solution with N_2_ bubbling: Before and after irradiation of Xe-290 nm light shown in Figure 3a, Figure S5: Photostability of **1** in toluene solutions with N_2_ bubbling using Xe-355 nm lamp, Figure S6: Triple decay emission at 77 K of **1**–**3** in toluene solutions, Figure S7: TCSPC time profile of **3** at 400 nm taken from Figure 6c, Figure S8: Time profiles of flu. (1), flu. (2) and phos. bands of **1**–**3** in toluene solutions taken from Figure 5, Figure S9: Optimized structure of **1–3** in the ground triplet state obtained by DFT calculations [M06-2X/6-31g(d)]. Table S1: Amplitudes (A_n_) and time constants (τ_n_) estimated by theoretical curve fittings of time-profiles of PL spectra of **1–3** in toluene solutions measured at 77 K, Table S2: TD-DFT calculation results of **1**−**3** with optimized conformation at the ground triplet state.
