# Supplementary material for: Excited-State Dynamics of Room-Temperature Phosphorescent Organic Materials Based on Monobenzil and Bisbenzil Frameworks
Source: Materials (Basel). 2020 Sep 3;13(17):3904. doi: 10.3390/ma13173904 (PMC7504296; doi:10.3390/ma13173904)
Supplement: Supplementary file 1 [file materials-13-03904-s001.pdf]

materials

Supplementary

Excited-State Dynamics of Room-Temperature Phosphorescent Organic Materials Based on Monobenzil and Bisbenzil Frameworks

Kaveendra Maduwantha[1,2], Shigeyuki Yamada [3], Kaveenga Rasika Koswattage [2,4], Tsutomu Konno [3] and Takuya Hosokai [2,*]

[1] Faculty of Graduate Studies, Sabaragamuwa University of Sri Lanka, P.O. Box 02, Belihuloya 70140, Sri Lanka; kavindrarox@gmail.com
[2] National Metrology Institute of Japan, National Institute of Advanced Industrial Science and Technology, 1-1-1 Higashi, Tsukuba 305-8565, Japan; koswattagekr@appsc.sab.ac.lk
[3] Faculty of Molecular Chemistry and Engineering, Kyoto Institute of Technology, Matsugasaki, Sakyo-ku, Kyoto 606-8585, Japan; syamada@kit.ac.jp (S.Y.); konno@kit.ac.jp (T.K.)
[4] Department of Engineering Technology, Faculty of Technology, Sabaragamuwa University of Sri Lanka, P.O. Box 02, Belihuloya 70140, Sri Lanka
* Correspondence: t.hosokai@aist.go.jp; Tel.: +81-29-861-1424

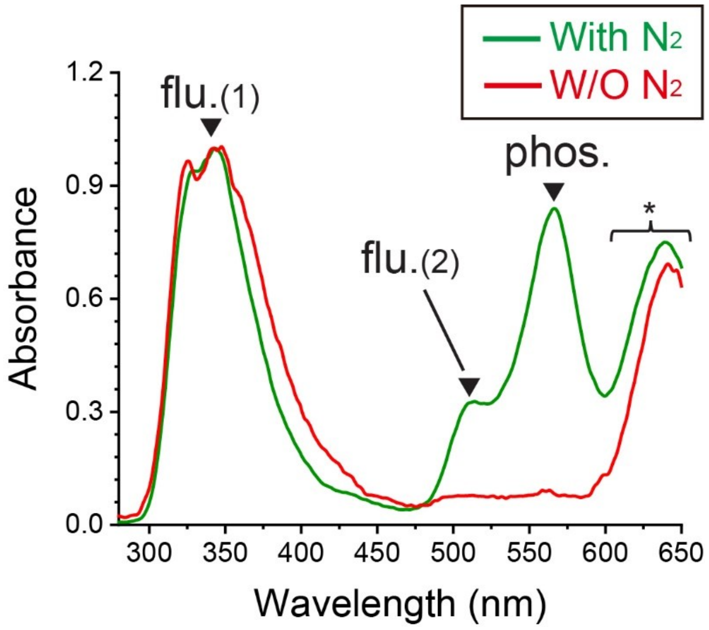

Figure S1. Steady state PL spectra of **1** at *t* = 0 min, taken from Figure 3a**.** Asterisk (*) is the region of second order diffracted light of flu. (1) band.

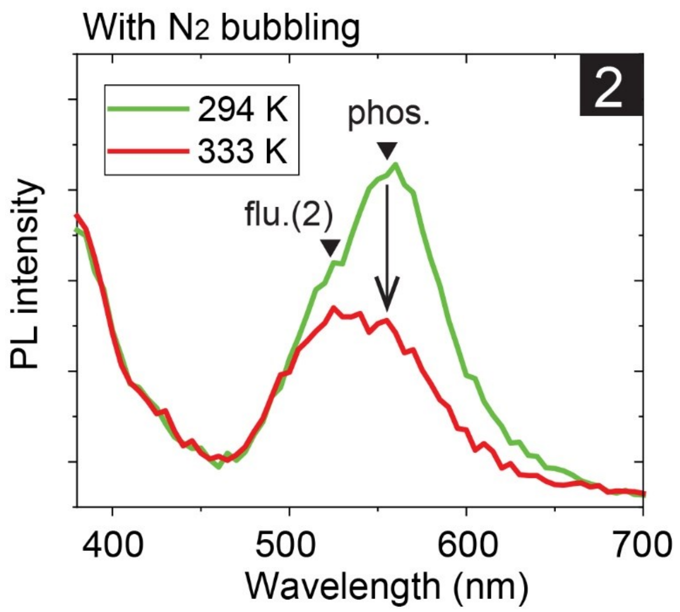

Figure S2. Temperature dependence of PL spectra of **2** after N_2 bubbling**.**

Khara et al. has reported that benzil in imidazolium ionic liquids showed two bands peaks at 505 nm and 560 nm, and an increase of the intensity for the 505 nm band measured at 10 msec at high temperatures [1]. They mentioned the high energy band is thermally activated delayed fluorescence (TADF) band, since TADF can be observed at the delay time over msec and the intensity is increased at higher temperature due to increase of the rate of reverse intersystem crossing. To investigate this point for our benzil molecules, we have also measured PL spectra at 294 and 333 K for **2** in toluene solution with N_2 bubbling. As shown in Figure S2, the flu. (2) band at around 505 nm does not increase at the higher temperature and instead phos. band decreases. This can be the indication of the high energy band for **2** not to be TADF. The decrease in the phos. band is caused by the increase of non-radiative decay rate from T_1 to S_0. Note that we confirm that such the intensity change was reversible when the temperature is returned to 294 K, which implies no deterioration in the measurement at the higher temperature.

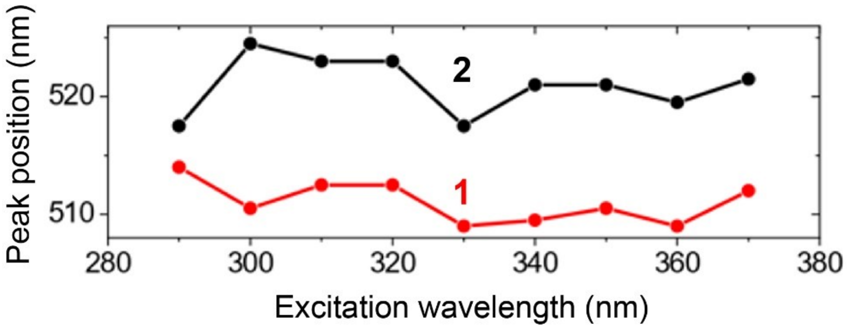

Figure S3. Excitation wavelength dependence of flu. (2) band peak of **1** and **2**. Standard deviation of the peak position is 1.8 nm for **1** and 2.4 nm for **2**.

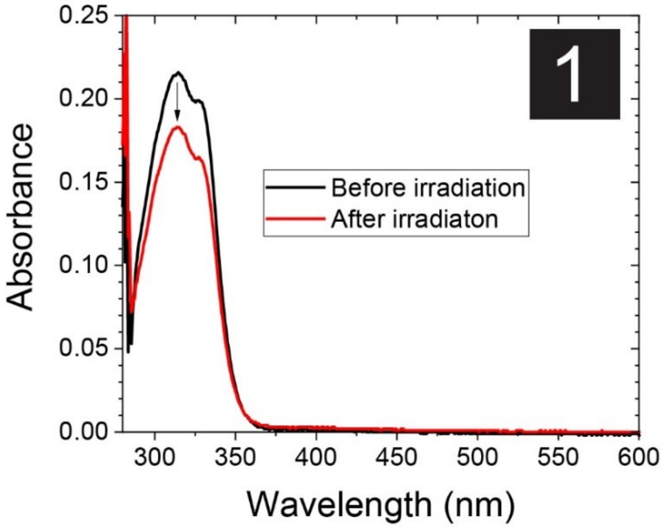

Figure S4. Absorptions spectra of **1** in toluene solution with N_2 bubbling: Before and after irradiation of Xe-290 nm light shown in Figure 3(a) in the main text.

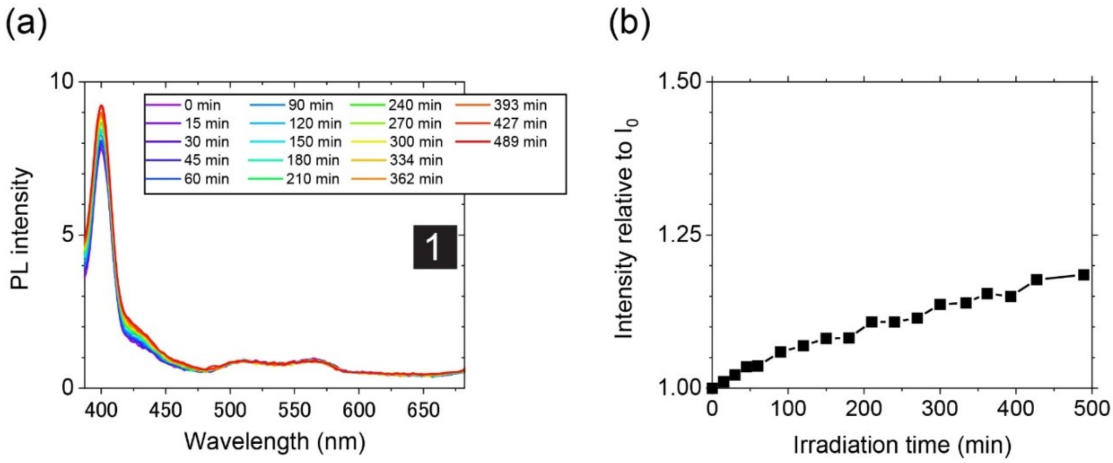

Figure S5. (**a**) Photostability of **1** in toluene solutions without N_2 bubbling using Xe-355 nm lamp. (**b**) The ratio of peak intensity of band at around flu.(1) band. I_0 is the initial peak intensity.

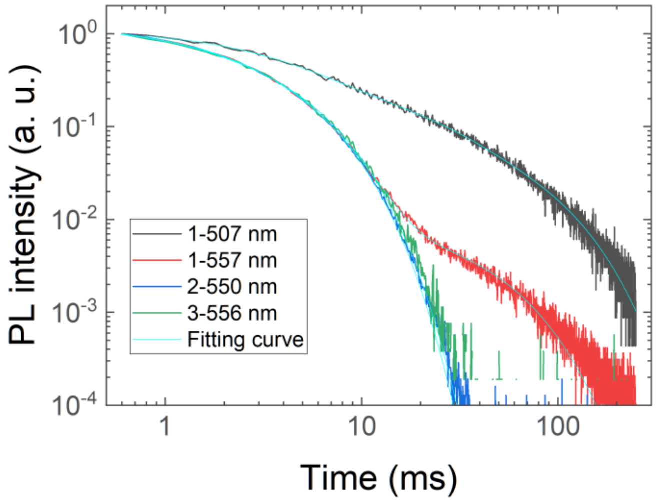

Figure S6. Triple decay emission at 77 K of **1**–**3** in toluene solutions. The peaks are chosen from Figure 4 in the main text.

Figure S6 shows the time profiles and theoretical curve fitting results of the T_1 and T_2 peaks for **1**–**3** by using multi-exponential decay functions, and the numerical results are summarized in Table S1. The time constant (τ) of phosphorescence from the common T_1 band (τ_1) is nearly the same as for all the **1**–**3** by 3.2 ~ 3.4 ms with the largest amplitude. For **2** and **3**, the second largest decay constant (τ_2) is shorter as 1.24 and 1.58 ms, is attributed to the initially observed spectral narrowing. For **1**, the double exponential decay function was required to fit the longer component of T_2 band, and the intensity averaged τ at 507 nm is estimated to 38.7 ms, one order of magnitude larger than the T_1 band.

Table S1. Amplitudes (A_n) and time constants (τ_n) estimated by theoretical curve fittings of time-profiles of PL spectra of **1**–**3** in toluene solutions measured at 77 K.

	A_1	τ_1 (ms)	A_2	τ_2 (ms)	A_3	τ_3 (ms)
**1@507 nm**	0.792	3.16	0.248	14.66	0.098	54.89
**1@557 nm**	0.789	3.20	0.482	1.58	0.010	33.67
**2@550 nm**	0.89	3.23	0.418	1.24	-	-
**3@556 nm**	0.88	3.37	0.434	1.07	-	-

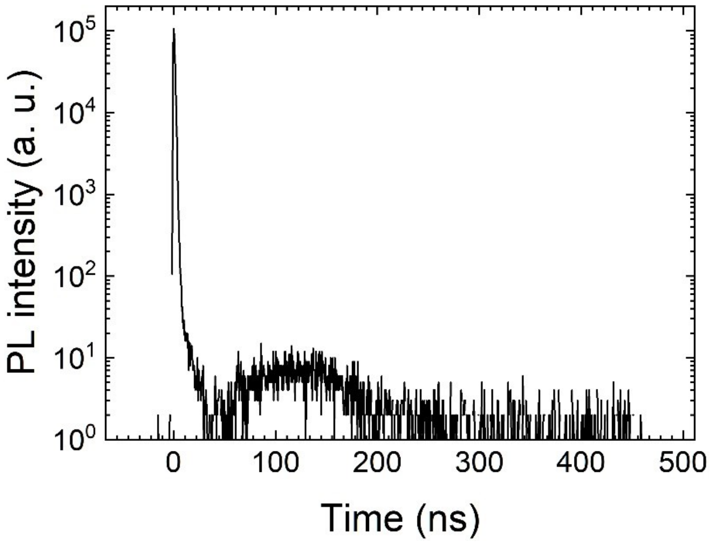

Figure S7. TCSPC time profile of **3** at 400 nm taken from Figure 6(c). The semilunar-like intensity from 30 to 200 ns is a characteristic of the measurement set-up, which made the artifact for the spectrum at 100 ns in Figure 6(c).

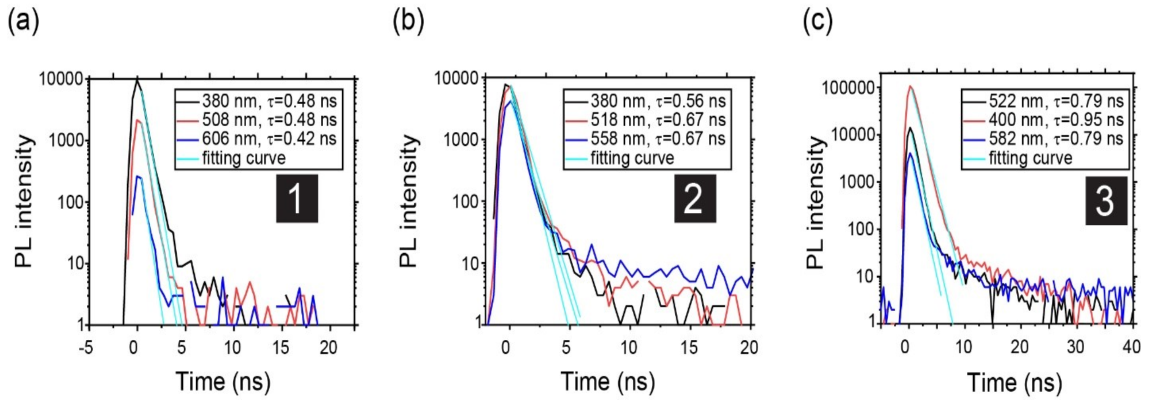

Figure S8. Time profiles of flu. (1), flu. (2) and phos. bands of **1**–**3** in toluene solutions taken from Figure 5.: flu. (1) is 380 nm for **1** and **2**, 400 nm for **3**, flu. (2) is 508 nm for **1**, 518 nm for **2**, and 522 nm for **3**, phos. is 606 nm for **1**, 558 nm for **2**, and 582 nm for **3**, respectively.

Table S2. TD-DFT calculation results of **1**–**3** with optimized conformation at S_1.

State	**1** (fluorinated monobenzil)		**2** (fluorinated bisbenzil)		**3** (non-fluorinated bisbenzil)	
	Excitation energy (eV) (Wavelength (nm))	Oscillator strength (f)	Excitation energy (eV) (Wavelength (nm))	Oscillator strength (f)	Excitation energy (eV) (Wavelength (nm))	Oscillator strength (f)
S_1	2.8359 (437.2)	0.0007	2.7781 (446.30)	0.0003	2.7955 (443.51)	0.0006
S_2	3.9664 (312.59)	0.002	2.7958 (443.46)	0.0004	2.8134 (443.46)	0.0002
S_3	4.1694 (297.36)	1.485	3.9273 (315.70)	0.0004	3.8911 (318.64)	0.0001
S_4	4.4886 (276.22)	0.066	3.9921 (310.57)	0.001	3.9381 (314.83)	0.0002
S_5	4.7753 (259.64)	0.0204	4.3241 (286.73)	0.3076	4.3978 (281.93)	0.387
S_6	4.8963 (253.22)	0.0012	4.5002 (275.51)	0.0354	4.5552 (272.18)	0.0353
S_7	5.0152 (247.22)	0.0034	4.7057 (263.48)	0.0218	4.7980 (258.41)	0.6492
S_8	5.0306 (246.46)	0.0176	4.753 (260.85)	0.6426	4.8850 (253.80)	0.0495
S_9	5.2327 (236.94)	0.0227	4.9721 (249.36)	0.014	5.0028 (247.83)	0.0146
S_{10}	5.2886 (234.44)	0.2488	5.0587 (245.09)	0.1495	5.0349 (246.25)	0.0339

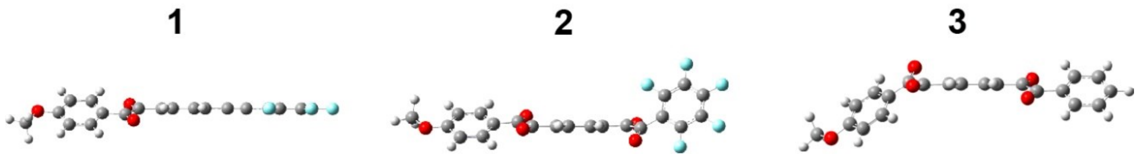

Figure S9. Optimized structure of **1**–**3** in the ground triplet state obtained by DFT calculations [M06-2X/6-31g(d)].

References

1. Khara, D.C.; Samanta, A. Fluorescence, Phosphorescence, and Delayed Fluorescence of Benzil in Imidazolium Ionic Liquids. *Aust. J. Chem.* **2012**, *65*, 1291–1297.
